# Elimination of *Tobacco rattle virus* from viruliferous *Paratrichodorus allius* in greenhouse pot experiments through cultivation of castle russet

**DOI:** 10.21307/jofnem-2020-011

**Published:** 2020-03-20

**Authors:** Richard A. Quick, Launa Cimrhakl, Hassan Mojtahedi, Vidyasagar Sathuvalli, Maximilian J. Feldman, Charles R. Brown

**Affiliations:** 1 Temperate Tree Fruit and Vegetable Research Unit, USDA-Agricultural Research Service, Prosser, WA; 2 Irrigation Agriculture Research and Extension Center, Washington State University, Prosser, WA; 3 AgNema LLC, Richland, WA; 4 Hermiston Agricultural Research and Extension Center, Oregon State University, Hermiston, OR

**Keywords:** Castle Russet, *Paratrichodorus allius*, Pathogenic virus, Potato, *Solanum tuberosum*, Stubby root nematode, *Tobacco rattle virus*

## Abstract

Corky ringspot (CRS) is a widespread potato tuber necrotic disease caused by *Tobacco rattle virus* (TRV) infection. In the Pacific Northwest, this virus is transmitted by the stubby root nematode (SRN) within the genus *Paratrichodorus*. Remediating CRS affected fields is a major challenge that can be mitigated by growing plant varieties that are resistant to TRV infection. Growing alfalfa has been shown to reduce TRV levels in CRS infested fields over time but the development of a potato cultivar with these same capabilities would be of great economic benefit to potato growers. Castle Russet is a new potato clone that does not develop symptoms of CRS disease. To assess its ability to reduce soil virus load, Castle Russet, tobacco var. “Samsun NN”, alfalfa var. “Vernema”, and Russet Burbank potato were grown for a period of 1 to 3 months in soils containing viruliferous SRN populations at two different inoculation pressures (60 nematodes/pot and 1060 nematodes/pot) in greenhouse pot experiments. SRN population size and the presence of TRV were assessed over several months post inoculation. Results indicate that plant host and length of exposure significantly influence SRN population dynamics, whereas the TRV infection status of bait plants was significantly affected by both of these factors as well as inoculation pressure. These results suggest that both alfalfa var. “Vernema” and Castle Russet are resistant to TRV infection and may potentially be used to eliminate the virus from fields affected by CRS.

Corky ringspot disease (CRS), also commonly referred to as “spraing” in Europe, is an important potato necrotic tuber syndrome caused by *Tobacco rattle virus* (TRV) infection. This viral pathogen is vectored by stubby root nematodes (SRNs) (*Paratrichodorus and Trichodorus* spp.) in the Trichodoridae family that reside in the soil as obligate ectoparasites (Robinson and Harrison, 1989). Symptoms of viral infection include the development of rust-colored necrotic lesions resembling concentric rings, linear arcs, or large diffuse spots within infected tubers. Economically, potatoes exhibiting CRS defects are categorized as culls by processors and fresh market purchasers causing devaluation or outright rejection of entire tuber lots if the proportion of affected tubers exceeds 6% (Ingham et al., 2000). The lack of visual foliar symptoms makes diagnosing infected seed tubers a serious problem for the seed certification agencies. Currently, there is no widely accepted method to test for TRV infection in seed tubers.

The spread of CRS is a widespread concern within the global potato industry. Both TRV and SRN co-occur throughout much of Europe (Hooker, 1981) and have been observed in more than ten states within the USA since first being observed in Florida in 1946 (Allen, 1963; David et al., 2010; Eddins et al., 1946; Gudmestad et al., 2008; Kirk et al., 2008; Thomas, 1976; Weingartner and Shumaker, 1990). Even small numbers of SRN (3 nematodes/250 cm^3^ of soil) can cause substantial CRS damage to a potato crop if left untreated (Mojtahedi et al., 2001). Mitigation of CRS in production settings is currently achieved by treating potato fields with expensive, non-selective pesticides that reduce the abundance of the SRN vector and planting virus insensitive cultivars (Yellareddygari et al., 2018) derived from seed growers’ fields that have been certified as being SRN and TRV free (Hafez and Sundararaj, 2009). Heavy usage of systemic pesticides is undesirable due to grower expense and negative impact on the environment; thus, development of potato cultivars that are resistant to TRV infection is an ideal solution. Breeding programs focused on this goal have developed and released several potato cultivars with TRV resistance (Brown et al., 2009, 2000; Dale, 1989; Dale and Solomon, 1988; Robinson and Dale, 1994; Shumaker et al., 1984).

Unfortunately, once a field has been affected by CRS, remediation is extremely difficult. Although bulk application of fumigants (1,3-dicholopropene, aka Telone II) and systemic nematicides (oxamyl, aka Vydate C-LV) can effectively reduce the population of the SRN vector in the soil, complete eradication is nearly impossible. SRN are mobile organisms that can survive deep in the soil profile re-emerging after pesticide application has subsided (Weingartner, 1983). Further complicating matters is the fact that TRV has one of the broadest host ranges of any virus (Boydston et al., 2004; Cooper and Harrison, 1973; Mojtahedi et al., 2003) and can lay dormant in weeds for years only to reappear when potato is cropped again.

Strategies to remediate land affected by CRS generally involve moving infected fields out of potato production and cropping varieties of alfalfa var. “Vernema” or Scotch spearmint var. “770” (Banttari et al., 1993; Mojtahedi et al., 2002) as rotation crops. These plant species are an adequate food source for SRN but cannot serve as hosts for TRV (Mojtahedi et al., 2002). Similar to other parasitic nematodes, SRN molts several times throughout its lifecycle, effectively shedding the virus with each molt (Robinson and Harrison, 1989). Greenhouse and field experiments suggest that if immature nematodes are not exposed to a virus-infected food source, the nematode population will no longer harbor the virus after several generations (Mojtahedi et al., 2002) and CRS symptoms will diminish (Banttari et al., 1993). Unfortunately, due to the specialized equipment needs and market factors, crop rotations that regularly include alfalfa and spearmint are not adequately profitable for potato growers.

Using greenhouse pot experiments, we have examined the potential of the TRV resistant potato cultivar, Castle Russet, to cleanse soils containing viruliferous SRNs of TRV infection relative to a known host (tobacco), a non-host (alfalfa), and an unrelated popular potato variety (Russet Burbank). The objectives of this study were to assess how host plant genotype, cultivation time, and nematode inoculation pressure influenced nematode fecundity and the presence of TRV through multiple generations of SRN.

## Materials and methods

### General information

Two independent experiments were carried out in a greenhouse at the USDA-ARS Temperate Tree Fruit and Vegetable Research Unit in Prosser, WA. The average temperature during the trials was 25°C with a 12-hour photoperiod. The plants were grown in 10 L plastic pots filled with a reconstituted sandy silt loam soil composed of 84% sand, 10% silt and 6% clay (Brown et al., 2006). The soil was steam pasteurized before use.

### Preparation of plant material and inoculum

For both experiments, tobacco var. “Samsun NN” seed was bulk sown into a 10 cm^2^ plastic pot filled with Sunshine #1 potting mix (Sun-Gro Horticulture Distributor Inc., Bellevue, WA) and plants were allowed to develop in a growth chamber (25°C; 12 h photoperiod). Alfalfa var. “Vernema” was direct seeded into Sunshine potting mix #1 in a 72-cell tray. In Experiment 1, plants of Russet Burbank and Castle Russet (aka POR06V12-3) were propagated first in tissue culture. The tissue culture media was prepared by dissolving in 1 L of double distilled water 4.41 g of MS Modified BC Potato Basal Medium, 8 g of Plant TC agar (Product Numbers M516 & A111, PhytoTechnology Laboratories Shawnee Mission, KS), and 25 g of sucrose. The mixture was autoclaved at 120°C for 20 min. Tissue culture plantlets were kept for 1.5 months in the tissue culture media to develop a massive root system, and were then transferred to a 72-cell tray in Sunshine potting mix # 1. The foliage was pruned to induce vigorous growth. In Experiment 2, disease-free nuclear tubers were produced from tissue cultured plants and stored at 5.5°C until the dormancy of the tubers was broken.

A viruliferous population of *Paratrichodorus allius* was maintained on TRV infected Samsun NN in a sandy silt loam soil (described above) in the greenhouse (24°C; 12 hr photoperiod). The identity of the nematode was confirmed using light microscopy after nematode extraction (Jenkins, 1964).

### Experimental design

**Figure 1:**
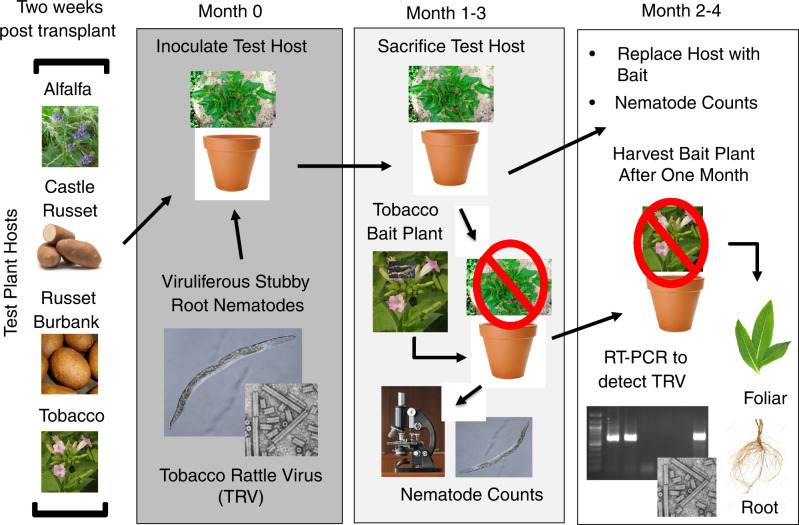
Two weeks post-transplant, plant hosts were inoculated to evaluate their ability to host Stubby root nematodes (SRN) and Tobacco rattle virus (TRV). At month zero, test hosts were transplanted into pots containing viruliferous SRNs and cultured for one to three months. Each month a subset of hosts is sacrificed, nematode counts are taken, and a tobacco bait plant is transplanted into the test pot. After one month the tobacco bait plant is harvested and both foliar and root tissue was tested for the presence of TRV using RT-PCR.

An overview of the experimental procedure can be observed in Figure 1. Experiment 1 was performed in April 2016. For this experiment, 10 tobacco, 30 alfalfa, 18 Russet Burbank and 28 Castle Russet were removed from the potting mix, roots washed, and then transplanted into pots containing the sandy silt loam soil. Two weeks after transplanting, 60 viruliferous *P. allius* in 5 mL of water were pipetted into three holes in each pot (~20 SRN/hole). Experiment 2 was conducted beginning in September 2016. In total, 15 tubers of Russet Burbank and Castle Russet were planted in early August 2016. Approximately four weeks post planting all plants had emerged; 5 tobacco and 15 alfalfa were transplanted from potting mix into the sandy silt loam described above. Two weeks post-transplant, 1,060 *P. allius* in 5 mL of water were pipetted into three holes in each pot (~350 SRN/hole). Subjects within each experiment were assigned treatment categories using a randomized block design.

One month post inoculation a number of replicates were destructively harvested and processed for evaluation of TRV infection. The number of replicates harvested was as follows: 10 tobacco, 10 alfalfa, 6 Russet Burbank and 6 Castle Russet for Experiment 1; 5 tobacco, 5 alfalfa, 5 Russet Burbank and 5 Castle Russet for Experiment 2. After plants were removed from the pots, a 250 cm³ sample of soil was collected and nematodes were isolated from the soil by sieving and sugar-centrifugal flotation (Jenkins, 1964). Nematodes were counted and returned to their original pots. Oostenbrink’s nematode reproduction factor (Rf=Final nematode count/Initial nematode population count) was calculated from these count numbers (Oostenbrink, 1966). Next, a bait plant (tobacco) was transplanted into the soil that remained in each of the harvested pots to ascertain if the nematodes remained viruliferous.

The harvested plants were washed and split into foliar, root and stolon/tuber (if present) sub-samples. For each sub-sample, approximately 0.5 to 1 g of tissue was grounded in grinding bags (Agdia, Inc., Elkhart IN). A total nucleic acid extraction was performed using a modified Dellaporta procedure (Crosslin and Hamlin, 2011). Nucleic acid pellets were resuspended in 400 μl of UltraPure distilled water (Invitrogen, Carlsbad, CA). The standardized RT-PCR reactions were conducted in 25 μl reactions that contained 12.5 μl of PCR mix (2× Reaction Mix from SuperScript® III One-Step RT-PCR System with Platinum® *Taq* High Fidelity DNA Polymerase kit, Life Technologies, Carlsbad, CA), 2.5 μl of RediLoad (Invitrogen), 0.5 μl of 20 μM solutions of each forward and reverse primers TRV 2/1, respectively (Crosslin and Hamlin, 2011), 6.5 μl UltraPure distilled water (Invitrogen), 0.5 μl of SuperScript® III Reverse Transcriptase Platinum *Taq* HiFi Mix (Life Technologies), and 2 μl of nucleic acid extracts. Thermal cycling (PTC200 thermal cycler, MJ Research) consisted of 15 min at 50°C, 5 min at 94°C, then 30 cycles of 15 sec at 94°C, 1 min at 58°C, 30 sec at 72°C, and a final incubation for 5 min at 72°C. Ten microliters of reaction products were resolved by electrophoresis in 1.5% agarose (LE, Analytical Grade, Promega, Madison, WI) gels in 10 mM Tris-acetate, 1 mM EDTA buffer (pH 8) for approximately 40 min at 100 V, stained with ethidium bromide, and observed under UV light.

Two months post inoculation, the above procedure was repeated for the following number of replicates: 10 alfalfa, 6 Russet Burbank and 6 Castle Russet for Experiment 1; 5 alfalfa, 5 Russet Burbank and 5 Castle Russet for Experiment 2. Here, plants were destructively harvested, the nematode Rf was calculated from soil samples, and new tobacco bait plants were added. In addition, the aforementioned bait plants were destructively harvested and evaluated for TRV infection by RT-PCR. Three months post inoculation, the above procedure was performed on the remaining replicates (10 alfalfa, 6 Russet Burbank and 16 Castle Russet) for Experiment 1; and (5 alfalfa, 5 Russet Burbank, and 5 Castle Russet) for Experiment 2. Again, plants were destructively harvested, the Rf was calculated from soil samples, and new tobacco bait plants were added. Additionally, the bait plants from the two-month evaluation were also tested. Four months post inoculation the bait plants from the three-month evaluation were destructively harvested and tested for TRV infection.

### Data processing and statistical analysis

All statistical analyses were performed within the R statistical computing environment (R Core Team, 2018). Generalized linear models were constructed to evaluate the influence of plant host (plant), exposure period (months), and inoculation pressure (experiment) on nematode count, Rf and TRV detection using RT-PCR. Nematode count was modeled as a negative binomial variable using the glm.nb function in the MASS library (Venables et al., 2002). Rf value was log transformed and modeled using the lm function in the base R package (R Core Team, 2018). To eliminate the confounding effects of zero values in log transformation an offset (half the minimum value found in the dataset) was added to all values. RT-PCR detection of TRV in bait plants was modeled as a binomial variable using the MASS library (Venables et al., 2002). The relationship between these variables was modeled using the following equation:

$$Y_{ijk}=\mu +{\mathrm{planthost}}_{i}+{\mathrm{month}}_{j}+{\mathrm{experiment}}_{k}+\varepsilon .$$

(1)

In which *Y*_*ijk*_ is the individual observation of the dependent variable, μ is the overall mean, plant host_*i*_ is the effect of the *i*th plant host, month_*j*_ is the effect of the *j*th month, experiment_*k*_ is the effect of the inoculation pressured applied in either of the *k*th experiment and *ε* is the random error term. The significance of each factor was tested using type III analysis of variance based upon likelihood ratio tests for the negative binomial and binomial variables (nematode count and RT-PCR detection of TRV respectively) and sum of squares in the case of log(Rf) using the car library in R (Fox and Weisberg, 2011).

For each characteristic (nematode count, Rf value, proportion of foliar and root TRV infection) groupings of genotype at the final sampling time point within each experiment were determined using Tukey’s honest significant distance (*α* < 0.05) as implemented using the agricolae package in R (de Mendiburu, 2019).

Raw data and analysis scripts used to generate tables and figures can be downloaded at the following Zenodo repository: https://doi.org/10.5281/zenodo.3710018.

## Results and discussion

### Population dynamics of stubby root nematodes

The capacity of tobacco var. Samsun NN, alfalfa var. “Vernema”, Russet Burbank potato and Castle Russet potato to sustain populations of viruliferous SRN was investigated in two independent greenhouse trials that differed by SRN inoculation pressure (Fig. 1). Overall, plant host (*χ* ^2^_host_ (3) = 50.14, *p*_host_< 7.45e^−11^) and exposure period (*χ* ^2^_month_ (1) = 26.09, *p*_month_< 3.27^−07^) both had a significant influence on SRN population size, whereas all three variables (plant host, exposure time and inoculation pressure) appeared to significantly affect the nematode Rf (*F*_host_ (3,175) = 21.25, *p*_host_ < 8.70^−12^; *F*_month_ (1,175) = 35.55, *p*_month_ < 1.34E^−08^; *F*_inoculation_ (1,175) = 6.76, *p*_innoculation_ < 0.002).

**Figure 2:**
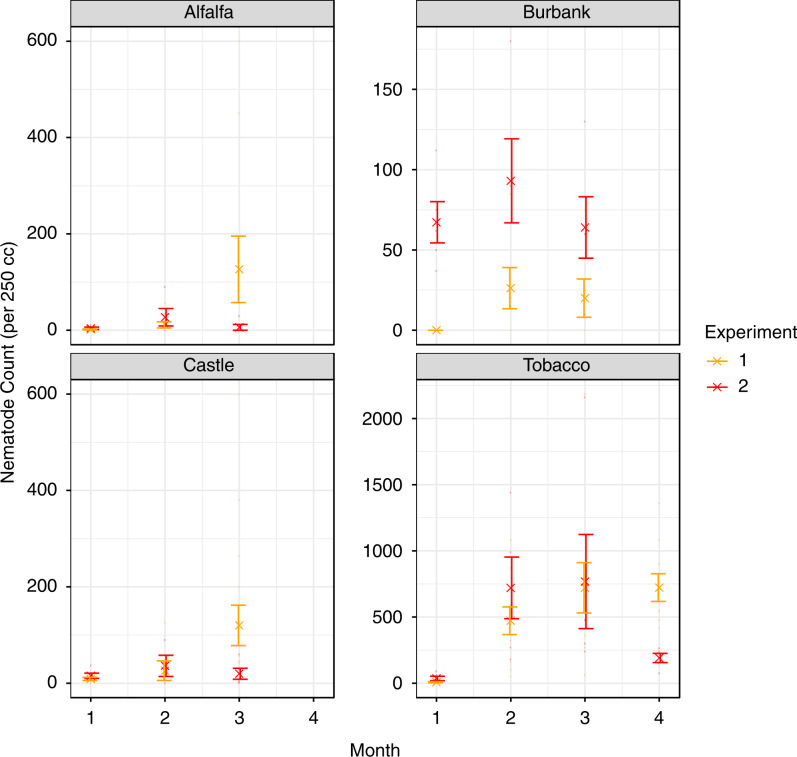
Population counts of stubby root nematode, *Paratrichodorus allius*, across three months in pots grown with alfalfa, Russet Burbank potato, Castle Russet potato and tobacco; inoculated with 60 and 1,060 nematodes/10 L soil in Experiment 1 and 2, respectively.

Statistics from both likelihood ratio *χ* ^2^ tests (SRN counts) and sum of squares estimation (Rf value) indicate plant host was the most influential factor, followed by the length of exposure, whereas inoculation pressure appeared to exhibit the smallest effect on both nematode count and Rf. Each plant species appeared to support increases of SRN populations throughout both experiments (Fig. 2 and Table 1) with Tobacco var. Samsun NN performing as the best plant host. Inoculation pressure significantly influenced Rf value after accounting for host plant and exposure time. Generally, mean Rf values were lower under the higher inoculation pressure applied in the second experiment (Fig. A1 and Table 1). In the first experiment, substantial reproductive population expansion occurred across all plant hosts and time points relative to the initial inoculum. This response was also observed when tobacco and Russet Burbank were used as host plants under the elevated inoculation pressure applied in the second experiment, whereas both alfalfa and Castle Russet did not exhibit a consistent increase relative to their initial inoculum. This may indicate that all four host plant genotypes can support growth of SRN populations, but Castle Russet and alfalfa do not allow SRN populations to increase without restriction. These results support the body of evidence that SRNs can reproduce on a broad range of host plants but with different efficacy (Ayala et al., 1970; Mojtahedi et al., 2003; Mojtahedi and Santo, 1999). Both cultivars of potato were poor hosts for the SRN but still maintained the capacity to support reproduction at levels higher than non-hosts including green pepper (*Capsicum annum*, California Wonder), carrot (*Daucus carota*, Chantenay), watermelon (*Citrullus vulgaris*, Charleston Gray), common groundsel (*Senicio vulgaris*), pigweed (*Amaranthus biltoides*, *A. powelli*, *A. retroflexus*), Coast fiddleneck (*Amsinckia intermedia*), Longsping sandbur (*Cenchrus longspinus*), Redstem filaree (*Erodium cicutarium*) and *Nicotiania clevelandi* (Mojtahedi and Santo, 1999; Mojtahedi et al., 2003).

**Table 1.** Average nematode count, reproduction factor (Rf) values and proportion of foliage and root tissue samples with TRV infection at the final sampling time point.

Plant	Experiment	Nematode count	Rf value	Foliage TRV infection (%)	Root TRV infection (%)
Alfalfa	1	126.40^b^	84.26^b^	0^a,b^	0^b^
Burbank	1	20.00^d^	13.33^b^	33^a,b^	50^a,b^
Castle	1	119.87^c^	79.91^b^	0^b^	0^b^
Tobacco	1	720.80^a^	480.53^a^	80^a^	100^a^
Alfalfa	2	6.00^d^	0.22^c^	0^b^	20^a^
Burbank	2	64^b^	2.41^b^	100^a^	100^a^
Castle	2	19.6^c^	0.73^b,c^	0^b^	0^a^
Tobacco	2	768^a^	28.98^a^	100^a^	80^a^

Notes: Experiment 1 was inoculated 60 nematodes/pot, whereas Experiment 2 was inoculated using 1,060 nematodes/pot. The final measurement of Nematode count and Rf value was performed after 3 months. The final measurement of TRV infection in foliage and root tissues was performed after 4 months. ^a,b,c,d^Superscripted letter assignments (a-d) reflect groupings determined using Tukey’s honest significant difference within the experiment (*α*<0.05).

### Prevalence of TRV infection

**Figure 3:**
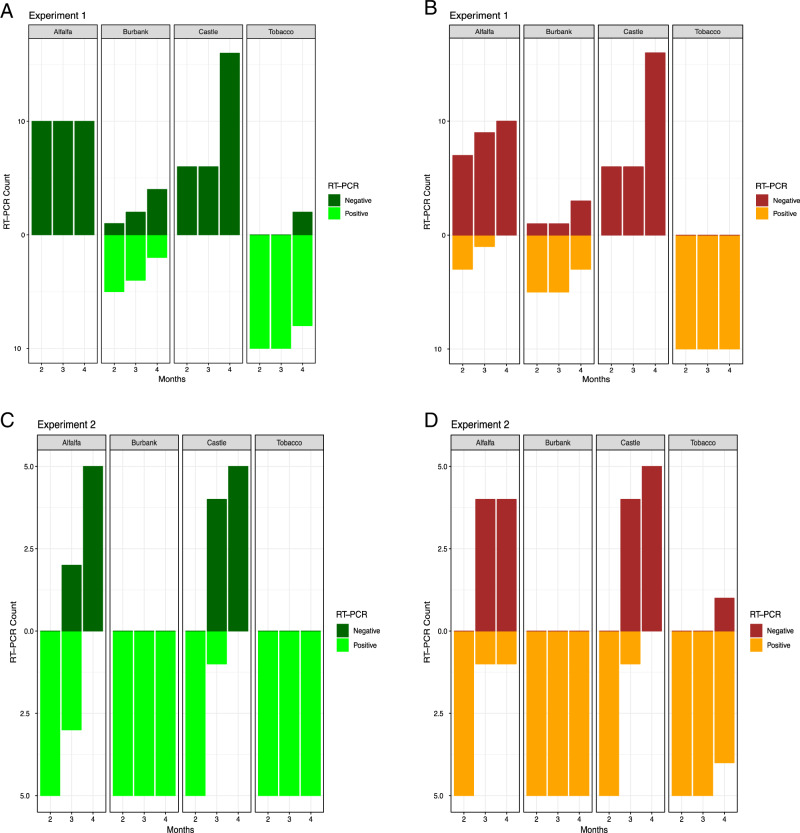
Number of bait plant tissue samples that tested positive and negative for the presence of TRV by RT-PCR. (A) Foliage tissue in Experiment 1 (B) Root tissue in Experiment 1 (C) Foliage tissue in Experiment 2 (D) Root tissue in Experiment 2.

The virulence of SRNs was evaluated over a three-month period by using RT-PCR to detect TRV from total nucleic acids extracted from foliage and root tissue of tobacco bait plants. The presence of TRV was significantly influenced by plant host (*χ* ^2^_root_ (3) = 130.22, *p*_root_ = 4.85e^−28^; *χ* ^2^_foliar_ (3) = 121.87, *p*_foliar_ = 3.05E^−26^), length of time cultured on the plant host (*χ* ^2^_root_ (1) = 25.92, *p*_root_ = 3.55e^−07^; *χ* ^2^_foliar_ (1) = 14.48, p_foliar_ = 0.00014), and the SRN inoculum pressure (*χ* ^2^_root_ (1) = 20.83, *p*_root_ = 5.03e^−06^; *χ* ^2^_foliar_ (1) = 40.32, *p*_foliar_ = 2.16e^−10^). Plant host was the most influential variable associated with infection status in both plant root and shoot tissue (*χ* ^2^_root_ (3) = 130.22, *p*_root_ = 4.85e^−28^; *χ* ^2^_foliar_ (3) = 121.87, *p*_foliar_ = 3.05E^−26^), whereas the length of exposure (*χ* ^2^_root_ (1) = 25.92, *p*_root_ = 3.55e^−07^) and inoculation pressure (*χ* ^2^_foliar_ (1) = 40.32, *p*_foliar_ = 2.16e^−10^) was the next most influential variable associated with TRV detection in the root and shoot tissue, respectively. As observed in previous studies (Mojtahedi et al., 2002), tobacco served as an excellent host for TRV and alfalfa exhibits substantial resistance to viral infection (Fig. 3). In the first experiment, tobacco bait plants transplanted into soil used to culture alfalfa exhibited zero TRV infection in foliar tissue and modest TRV infection in root tissue (30% after two months, 10% after three months) that decreased to zero percent infection after four months. Under high inoculation pressure, a notable percentage of bait plants cultured in alfalfa soil exhibited TRV infection in root and foliar tissue (Fig. 3). However, the proportion of TRV infected bait plants decreased relative to the length time alfalfa was grown in the test soil supporting the evidence that alfalfa is capable of reducing the viral load present in the soil (Mojtahedi et al., 2002). Russet Burbank potato behaved as a poor host for TRV in the first experiment. Tobacco bait plants transplanted into soil previously containing Russet Burbank potato were able to contract TRV in the first experiment although the fraction of infected foliar tissue samples decreased proportionally with the length of time Russet Burbank was grown. When exposed to the high inoculation pressure of the second experiment, tobacco bait plants grown in Russet Burbank potato soil displayed 100% TRV infection in both foliar and root tissues. Castle Russet potato exhibited almost complete resistance to TRV infection. No TRV was detected in either foliar or root tissue in tobacco bait plants transplanted into soils that previously contained Castle Russet potato in the first experiment. Under higher inoculation pressure applied in the second study, the percentage of infected material detected by RT-PCR decreased as the length of time Castle Russet potato was grown prior to transplant increased (Fig. 2C-D). These results indicate that Castle Russet, like alfalfa and scotch spearmint, is resistant to TRV infection (Mojtahedi et al., 2002). The mechanism of TRV viral load reduction is likely due to loss of TRV from the SRNs as part of the molting process (Ayala and Allen, 1968; Macfarlane, 2003). Based upon these results, we speculate that cultivation of Castle Russet in rotation with other TRV non-host crops (alfalfa, scotch spearmint) may provide potato growers with an important tool to maintain profitability while simultaneously reducing viral load in potato fields affected by CRS.

## Figures and Tables

**Figure A1:**
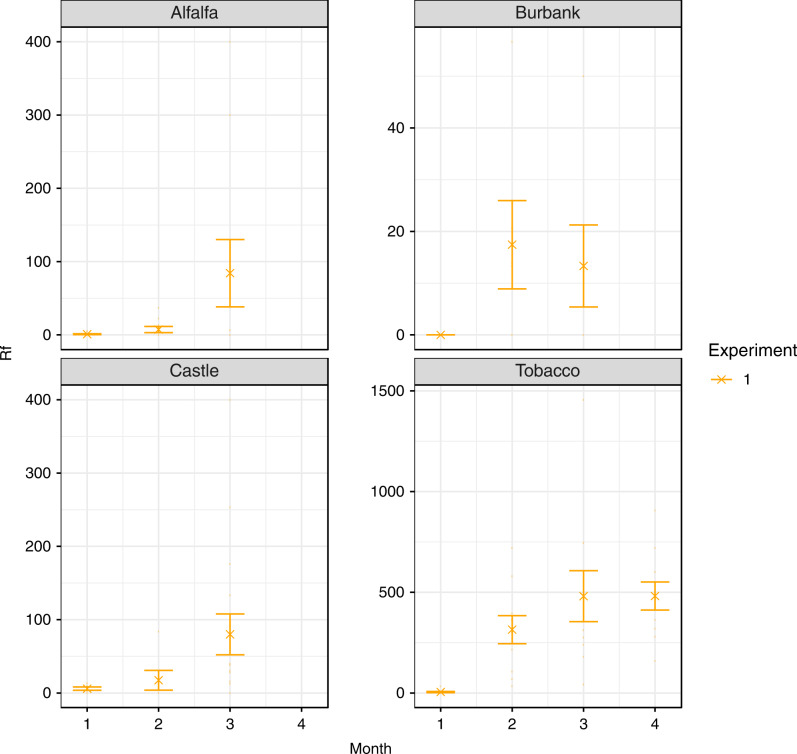
Nematode reproduction factor (Rf) estimates calculated from 250 cm^3^ soil samples collected from four plant genotypes, across three months, in two independent experiments. Individual data points are plotted as circular dots, whereas the population mean is plotted using an “X” symbol and standard error of the mean is denoted by the error bars. Experiments are differentiated by inoculation pressure. (A) Experiment 1 (60 nematodes/10 L) is plotted in orange (B) Experiment 2 (1,060 nematodes/10 L) plotted in red.

**Figure A1:**
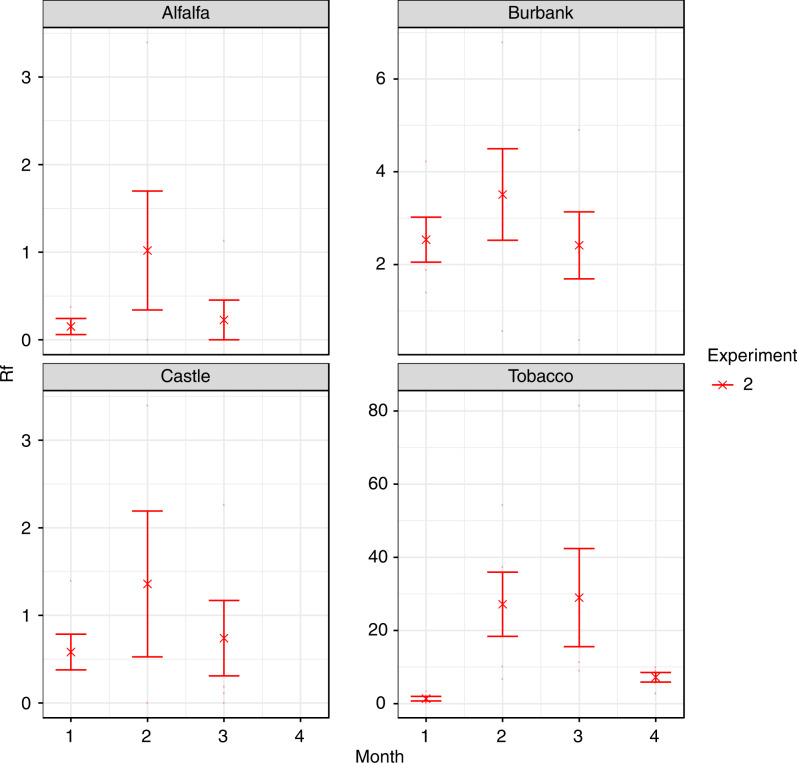
(continued)
